# Rolling Bearing Fault Diagnosis Based on Markov Transition Field and Residual Network

**DOI:** 10.3390/s22103936

**Published:** 2022-05-23

**Authors:** Jialin Yan, Jiangming Kan, Haifeng Luo

**Affiliations:** 1 School of Technology, Beijing Forestry University, Beijing 100083, China; yanjialin@bjfu.edu.cn (J.Y.); kanjm@bjfu.edu.cn (J.K.); 2 Key Laboratory of State Forestry Administration on Forestry Equipment and Automation, Beijing 100083, China

**Keywords:** intelligent fault diagnosis, Markov transition field, residual network

## Abstract

Data-driven rolling-bearing fault diagnosis methods are mostly based on deep-learning models, and their multilayer nonlinear mapping capability can improve the accuracy of intelligent fault diagnosis. However, problems such as gradient disappearance occur as the number of network layers increases. Moreover, directly taking the raw vibration signals of rolling bearings as the network input results in incomplete feature extraction. In order to efficiently represent the state characteristics of vibration signals in image form and improve the feature learning capability of the network, this paper proposes fault diagnosis model MTF-ResNet based on a Markov transition field and deep residual network. First, the data of raw vibration signals are augmented by using a sliding window. Then, vibration signal samples are converted into two-dimensional images by MTF, which retains the time dependence and frequency structure of time-series signals, and a deep residual neural network is established to perform feature extraction, and identify the severity and location of the bearing faults through image classification. Lastly, experiments were conducted on a bearing dataset to verify the effectiveness and superiority of the MTF-ResNet model. Features learned by the model are visualized by t-SNE, and experimental results indicate that MTF-ResNet showed better average accuracy compared with several widely used diagnostic methods.

## 1. Introduction

Rolling bearings are critical components in rotating machinery, and their operating conditions under various loads directly impact their performance, stability, and endurance. More specifically, rolling bearings are vital in mechanical equipment. To maintain the normal operation of mechanical equipment, it is necessary to monitor the vibration signals generated by the rotating mechanism in real time [1]. Many scholars extensively studied the fault detection and diagnosis of rolling bearings [2,3,4]. The traditional manual diagnostic can no longer adapt to the large-capacity, diverse, and high-speed data in the current mechanical field, which leads to poor diagnosis capability and generalization performance in the face of massive amounts of mechanical equipment data with alternating multiple working conditions and the serious coupling of fault information [5].

The diagnosis of rolling bearings generally consists of two stages: feature extraction and classification. Signal processing approaches that are widely employed to extract features from a raw signal include short-time Fourier transform (STFT) [6], wavelet transform (WT) [7], and empirical mode decomposition (EMD) [8]. However, traditional fault diagnosis methods rely heavily on manual feature engineering and expert knowledge, and the process is time-consuming and laborious. In addition, when extracted features are insufficient, the accuracy of fault diagnosis is greatly reduced, which is not conducive to the diagnostic tasks of massive amounts of industrial data. In the past decade, machine-learning theories and statistical inference techniques have been widely applied to identify bearing faults, such as Bayesian networks [9], artificial neural networks (ANNs) [10], support vector machines (SVMs) [11], and k-nearest neighbor [12]. Despite the effectiveness of the above-mentioned methods, shallow networks are restricted in their capacity to represent complicated functions with limited samples; thus, they lack the ability to diagnose the faults of complex and high-dimensional signals.

In recent years, deep-learning models have grown in popularity in the field of machine learning, which uses the deep network structure to achieve more efficient and reliable feature extraction. Deep learning disposes of the dependence on manually extracting features and expert experience, which has achieved breakthroughs in many pattern recognition tasks such as natural-language processing [13], automatic speech recognition [14], and computer vision [15]. The application of deep-learning models in fault diagnosis and health monitoring is flourishing [16,17]. Shao et al. [18] proposed a new deep belief network, which was optimized with the particle swarm algorithm, and verified the robustness of the model. Wen et al. [19] developed a novel DTL model for fault diagnosis that extracted features with a three-layer sparse autoencoder and achieved high prediction accuracy. Jiang et al. [20] constructed a deep recurrent neural network with an adaptive learning rate for the fault diagnosis of bearings, and results confirmed the effectiveness of the method. Hasan et al. [21] proposed an explainable AI-based fault diagnosis model and incorporated explainability to the feature selection process. Within the deep-learning framework, convolutional neural networks, as an end-to-end learning model with powerful feature extraction capability, have received more attention in fault diagnosis. Chen et al. [22] developed bearing discrimination patterns on the basis of the cyclic spectral coherence (CSCoh) maps of vibration signals and established a CNN model to learn high-level features. Guo et al. [23] proposed a new method named DCTLN for transfer fault diagnosis tasks, and verified the effectiveness of the model by experiments. Jia et al. [24] proposed a DNCNN to address imbalanced classification problems in fault diagnosis. In some scenarios, raw one-dimensional signals are converted into two-dimensional gray images with pixels fulfilled by data stacking [25,26]. However, these methods may contain limited feature information because spatial correlation in a raw vibration sequence can be corrupted. Although there are a few commonly used image representation approaches based on time–frequency principles, such as short-time Fourier transform (STFT) [6] and wavelet packet transform (WPT) [27], short-time Fourier transform is not suitable for handling nonstationary signals such as mechanical fault signals, and the determination of the number of decomposition layers for wavelet packets usually relies heavily on expert knowledge. Therefore, a new image encoding method called Markov transition field (MTF) was introduced [28] that preserves complete time-domain information by representing Markov transition probabilities, and converts that information into two-dimensional images. In addition, despite the great success of deep convolutional neural networks, degradation problems such as gradient disappearance or explosion can occur as the number of layers increases. To address the issue mentioned above, He et al. [29] proposed residual networks that have achieved excellent performance on various machine-learning tasks.

In order to efficiently represent the state characteristics of vibration signals in image form and improve the feature learning capability of the network, a new intelligent bearing fault diagnosis method (MTF-ResNet) is proposed in this paper. The main contributions of this paper are summarized as follows.

1. A novel two-step fault diagnosis method is proposed that converts raw vibration signals into images through the Markov transition field, and adopts the residual network for feature extraction and fault identification.
2. The signal-to-image conversion preserves the time dependence of the raw vibration signals and retains sufficient temporal features without setting parameters involving expert knowledge. Residual learning is applied to effectively address degradation problems in the deep neural network.
3. The effectiveness of the proposed model was verified on a popular bearing dataset. Compared with some existing methods, the MTF-ResNet method achieved better accuracy in bearing fault diagnosis.
4. To further demonstrate the performance of the proposed method and investigate the intrinsic mechanism of the CNN model in bearing fault diagnosis, t-SNE was used to visualize the feature maps learned by the model.

The remainder of this paper is organized as follows. Section 2 introduces the fundamentals of CNN and residual networks. In Section 3, the details of the proposed MTF-ResNet model for fault diagnosis are elaborated. Section 4 outlines experimental analysis to verify the effectiveness of the proposed model by employing a popular bearing dataset. Section 5 presents the conclusions.

## 2. Background and Related Work

Motivated by the concept of various cells in the visual cortex of the brain, and some cells that are exclusively responsive to the local receptive field [30], convolutional neural networks (CNNs) were first proposed by LeCun [31] for image processing. A typical CNN involves three different layers: (1) convolutional layer, (2) subsampling or pooling layers, and (3) fully connected layer. The convolutional layer comprises a number of kernels that extract features from input data. The pooling layer is the downsampling layer to reduce the trained parameters. The fully connected layer is a traditional feed-forward neural network where all neurons are connected to the activation of the previous layer. In this section, we describe CNNs and residual networks in more detail.

### 2.1. Convolutional Layer

The convolutional layer performs convolutional operations on local regions of the input data (or features) with the use of the convolutional kernel. Weight sharing is the most essential characteristic of the convolutional layer, since the input is traversed once by the same convolutional kernel at a set stride which can minimize the parameters and alleviate overfitting to some extent. In general, the mathematical model of the convolutional layer can be described as:

(1) $$x_{j}^{l}=\sigma (\sum {}_{i\in M_{j}}x_{j}^{l-1}*k_{ij}^{l}+b_{j}^{l})$$

where $x_{j}^{l-1}$ is the input to the (𝑙 − 1)st layer of the network; $x_{j}^{l}$ is the output of layer 𝑙 of the network; $k_{ij}^{l}$ is the weight matrix of the convolution kernel; $b_{j}^{l}$ is the bias; $M_{j}$ denotes the set of input feature maps; σ represents the nonlinear activation function; ∗ represents the operation of convolution.

### 2.2. Pooling Layer

The main function of the pooling layer is to reduce the dimensionality of the data after convolutional operations. Average and maximal pooling are two commonly used pooling methods. The pooling layer performs a downsampling operation on the feature map, which avoids overfitting to a certain extent while retaining key features. The pooling layer transformation can be described as:

(2) $$x_{j}^{l}=\sigma (\beta_{j}^{l}down(x_{j}^{l-1})+b_{j}^{l})$$

where down(⋅) represents the downsampling function, $\beta_{j}^{l}$ is the multiplicative weight.

### 2.3. Residual Network

Traditional deep convolutional neural networks are difficult to train as the network deepens because of problems of vanishing and exploding gradients. To address the degradation problem, He et al. [29] proposed deep residual networks that are widely used in image processing. The structure of the residual networks is shown in Table 1.

**Figure 1:**
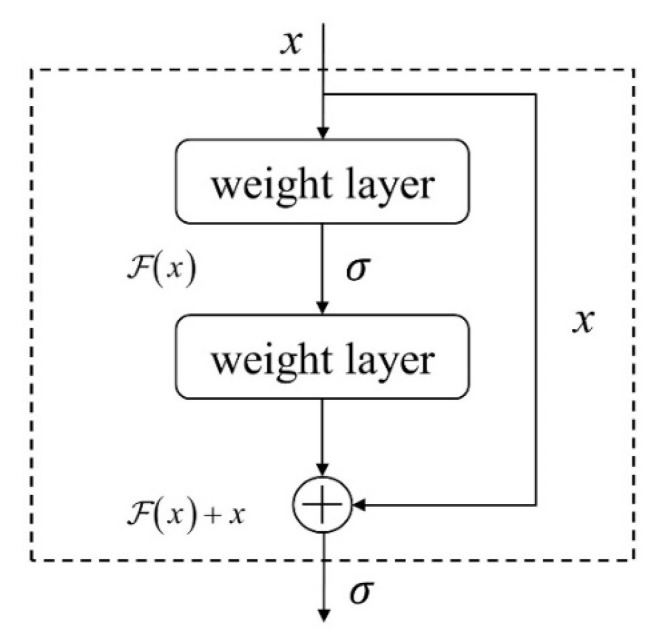
Residual building block.

Residual building blocks are the basic components of a residual network. As shown in Figure 1, a residual building block is composed of several convolutional layers, batch normalizations (BNs), ReLU activation functions, and an identity shortcut. The residual building block can be expressed as:

(3) $$y=ℱ(\mathrm{x,}\{W_{i}\})+x$$

where x represents the input vectors of the layer and y represents the output. $ℱ(x,\{W_{i}\})$ denotes the residual mapping function. Take the diagram in Figure 1 for example, $ℱ=W_{2}\sigma \left(W_{1}x\right)$, where σ denotes the nonlinear activation function (ReLU).

## 3. Proposed Model for Fault Diagnosis

This section presents the proposed MTF-ResNet fault diagnosis method. First, data augmentation is used to increase the training data. Then, the conversion method of the vibration signals into images is presented. Lastly, the network architecture based on MTF and ResNet for rolling bearing fault diagnosis is established.

### 3.1. Data Augmentation

**Figure 2:**
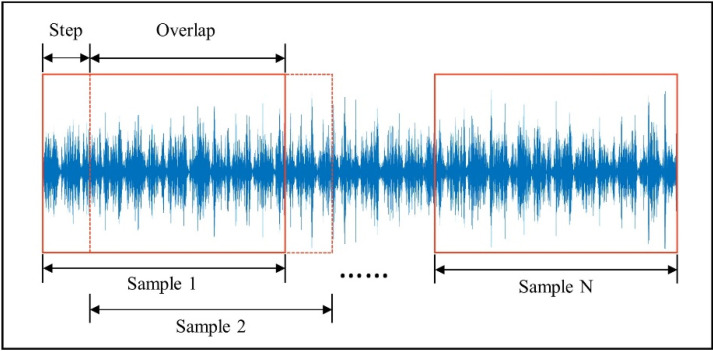
Process of data augmentation.

An effective technique to improve the generalization capabilities of machine-learning models is to use additional training samples. In computer vision tasks, horizontal flips, random crops or scales, and color jitter are commonly utilized to increase the data to train the model. Data augmentation is also required in fault diagnosis for deep convolutional neural networks to achieve high classification accuracy and avoid overfitting. The data augmentation method used in this paper is overlapping samples from raw one-dimensional sequences. Augmented samples were all allocated the same fault label as that of the raw sequence, since each input sequence was obtained under a single fault state. The data augmentation process is shown in Figure 2. The specific calculation method is expressed as follows:

(4) $$N=\frac{L-l}{s}+1$$

where L is the length of the raw signal, l is the length of a single sample, s is the shift stride, and N is the number of samples obtained through data augmentation.

### 3.2. Signal-to-Image Conversion

When diagnosing and analyzing bearing faults, the accelerometer is one of the most frequently used sensors in modern research, which can directly collect the original vibration signal of the target object. Collected data from industrial processes are continuous time series, and have the characteristics of nonlinearity and nonstationary caused by high coupling in the system.

Assume a time series $X=\{x_{1},x_{2},\cdots ,x_{n}\}$; the values can be quantized in Q bins, and each $x_{i}$ can be allocated to a related $q_{j}(j\in [1,Q])$. By calculating the transitions among bins in the way of a first-order Markov chain along each time step, a matrix W of Q×Q size is obtained. $w_{i,j}$ is the probability that an element in $q_{j}$ is followed by an element in $q_{i}$. After normalization by $\sum_{j=1}^{Q}w_{ij}=1$, W is considered to be the Markov transition matrix. Since the matrix is not sensitive to the distribution of X and time steps $t_{i}$, in order to reduce the loss of information, the $M_{ij}$ in the Markov transition field (MTF) is defined as follows:

(5) $$M_{ij}=\left[\begin{array}{ccc} w_{ij}|x_{1}\in q_{i},x_{1}\in q_{j} & \cdots & w_{ij}|x_{1}\in q_{i},x_{n}\in q_{j}\\ w_{ij}|x_{2}\in q_{i},x_{1}\in q_{j} & \cdots & w_{ij}|x_{2}\in q_{i},x_{n}\in q_{j}\\ \vdots & \ddots & \vdots \\ w_{ij}|x_{n}\in q_{i},x_{1}\in q_{j} & \cdots & w_{ij}|x_{n}\in q_{i},x_{n}\in q_{j} \end{array}\right]$$

The Markov transition field (MTF) then can be defined as follows:

(6) $$M=\left[\begin{array}{ccc} M_{11} & \cdots & M_{1n}\\ M_{21} & \cdots & M_{2n}\\ \vdots & \ddots & \vdots \\ M_{n1} & \cdots & M_{nn} \end{array}\right]$$

$M_{ij}$ is the probability that an element in $q_{j}$ is followed by an element in $q_{i}$. In other words, MTF incorporates temporal information on the basis of the Markov transfer matrix and actually represents the multispan transition probabilities of the time series. Such an expansion can denote not only the state transition for a single time stamp i. but also characterize state transitions over multiple time bins according to changes in the elements of MTF. $M_{ij\left\|i-j\right\|=k}$ represents the transition probability between points with a time interval k. A special case is that, when k=0, main diagonal $M_{ii}$ obtains the probability from each quantile to itself at time step i.

**Figure 3:**
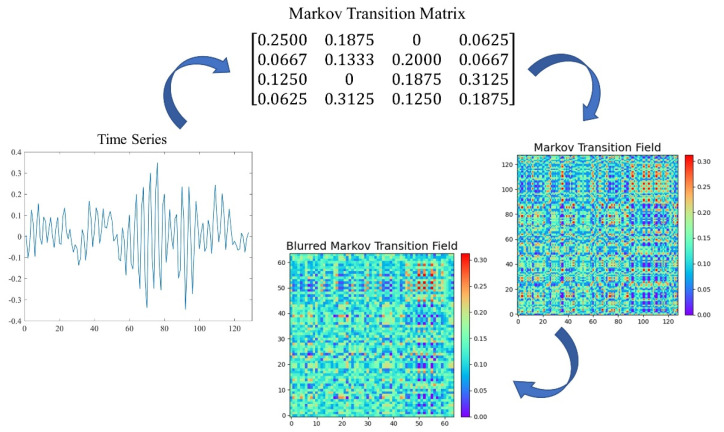
Transformation process of Markov transition field.

In the MTF matrix, the $M_{ij}$ can be regarded to be a pixel point represented through the colormap. Red denotes a larger value, while blue denotes a smaller value. It is inappropriate to directly employ images generated by MTF as the input of CNN since the images may be too large for training the model. In order to reduce the size of the images and improve computation efficiency, blurring kernel ${\left\{\frac{1}{m^{2}}\right\}}_{m\times m}$ was adopted to average the pixels in each nonoverlapping m×m region. The transformation process of the Markov transition field is shown in Figure 3.

### 3.3. Network Architecture

**Figure 4:**
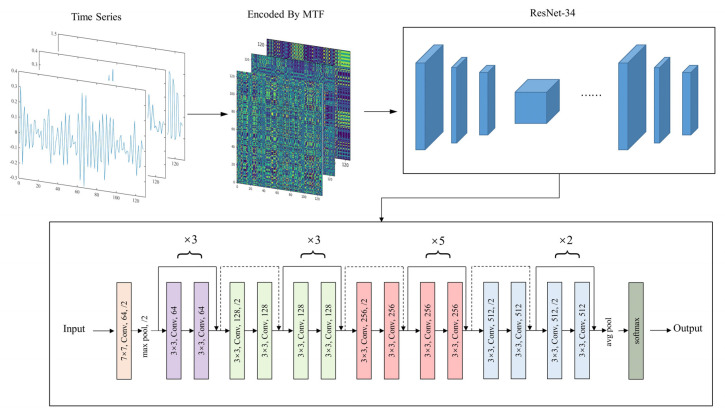
Architecture of the proposed MTF-ResNet model.

Once the raw vibration signals are converted into MTF images and formed into the image dataset, a CNN model can be trained to classify these images. In this paper, we applied the ResNet-34 network to extract 2D image features. A softmax layer was employed at the end of the network to classify the rolling-bearing health condition on the basis of the learned features. The proposed MTF-ResNet model architecture is demonstrated in Figure 4. The detailed parameters of the MTF-ResNet model are presented in Table 2.

## 4. Experiments and Results

### 4.1. Data Processing

**Figure 5:**
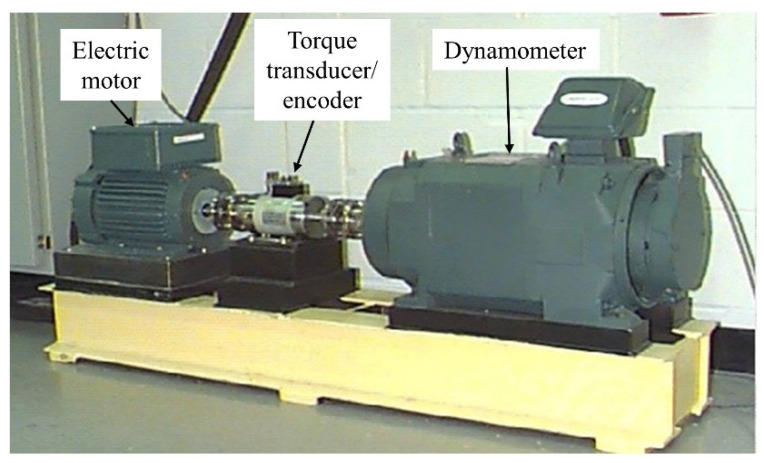
CWRU bearing test rig [32].

To validate the performance of the proposed MTF-ResNet, the Case Western Reserve University (CWRU) [32] bearing dataset was employed to conduct experiments. The test rig comprised an electric motor, a torque transducer/encoder, and a dynamometer, as shown in Figure 5. The bearing to be tested rotatably supports the shaft of the motor under four load conditions: 0, 1, 2 and 3 hp with motor speeds of 1772, 1750, and 1730 r/min. Different types and severity levels of bearing failures are caused by the use of electrical discharge machining (EDM), including normal condition (NC), inner-race fault (IF), outer-race fault (OF), and rolling ball fault (BF). For each fault state, three kinds of fault diameters were set: 0.007, 0.014, and 0.021 inches, respectively.

In this paper, we used raw vibration signal sample at 12 kHz from the drive end accelerometer (DE). The training data were generated from half of the raw vibration sequence by overlapping samples through a sliding window length of 2048 with a step size of 80, while the test data were generated by the same window length from the other half without data augmentation. According to the working conditions, datasets under a single working condition and variable working conditions are considered in this study. The bearing fault datasets under a single working condition are shown in Table 3; each dataset contained 6600 training samples and 250 testing samples from 10 fault types, as presented in Table 4. The composition of bearing fault data under variable working conditions is shown in Table 5.

**Figure 6:**
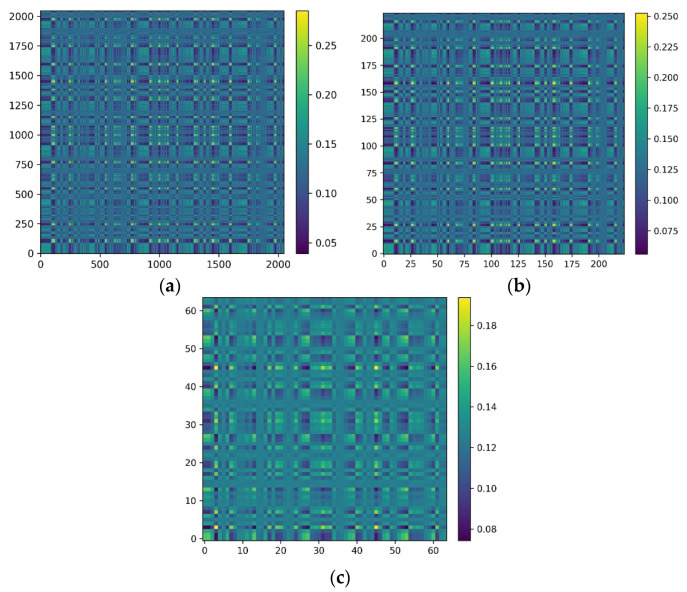
Transformation of the same signal containing 2048 data points into MTF images of image sizes of (**a**) 2048 × 2048, (**b**) 224 × 224, and (**c**) 64 × 64.

All samples were then converted into MTF images. Figure 6 shows the transformation of the same signal containing 2048 data points into MTF images of different image sizes. Large MTF images generally result in an increase in computational cost and are not conducive to the training of the model. However, small MTF images can hardly contain enough useful information. On the basis of the above considerations, the size of the MTF images was determined to be 224 × 224.

### 4.2. Data Analysis

**Figure 7:**
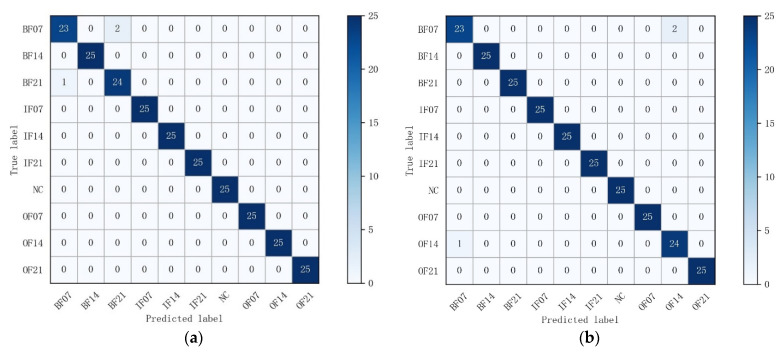

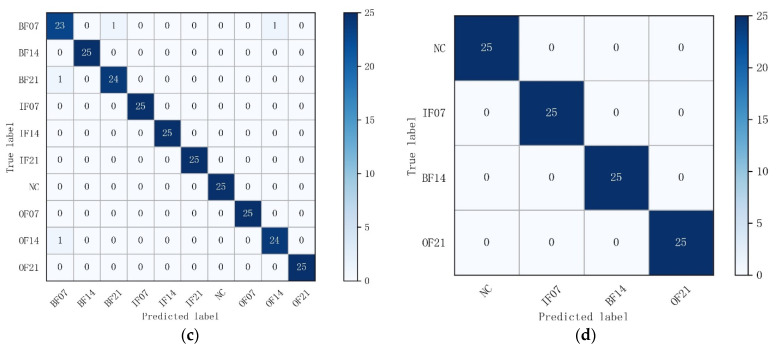
Confusion matrixes for each dataset: (**a**) Dataset A; (**b**) Dataset B; (**c**) Dataset C; (**d**) Dataset D.

In order to show the detailed identification effect of the model for each fault type in the test set, a confusion matrix was introduced for more accurate and comprehensive analysis of the experimental results. The vertical axis of the confusion matrix represents the true labels of the classification, and the horizontal axis demonstrates the predicted labels. The confusion matrix shows the classification results of all fault types, containing both correct and incorrect classification information. The confusion matrices of the MTF-ResNet prediction results are shown in Figure 7. In Dataset A, there was a slight error in the classification of fault types BF07 and BF21, two samples of bearing fault type BF07 were incorrectly labeled as BF21, and one sample of BF21 was identified as BF07; all other samples were correctly classified by the MTF-ResNet model. In Dataset B, the incorrect classification occurred in the identification of BF07 and OF14, two samples with the true label BF07 were incorrectly mistaken for OF14, and one sample belonging to the OF14 fault type was classified as BF07, the model achieved correct classification in all other fault types. In Dataset C, the situation was similar to that in Datasets A and B: two samples in BF07 were identified as BF21 and OF14, while one sample in each of BF21 and OF14 was misclassified as BF07. Samples of all fault types were correctly identified by the model in Dataset D. The accuracy of the model in Datasets A–D was 98.8%, 98.8%, 98.4%, and 100%, respectively. It is clear from the experimental results that almost all of the misclassifications occurred in the diagnosis of ball faults, which coincides with the findings in [32] that there are undiagnosed outer and inner race faults in the drive end bearing, probably caused by brinelling. We conducted several trials, and the average accuracy of the model in the 10- and 4-category datasets was 98.52% and 100%, respectively.

**Figure 8:**
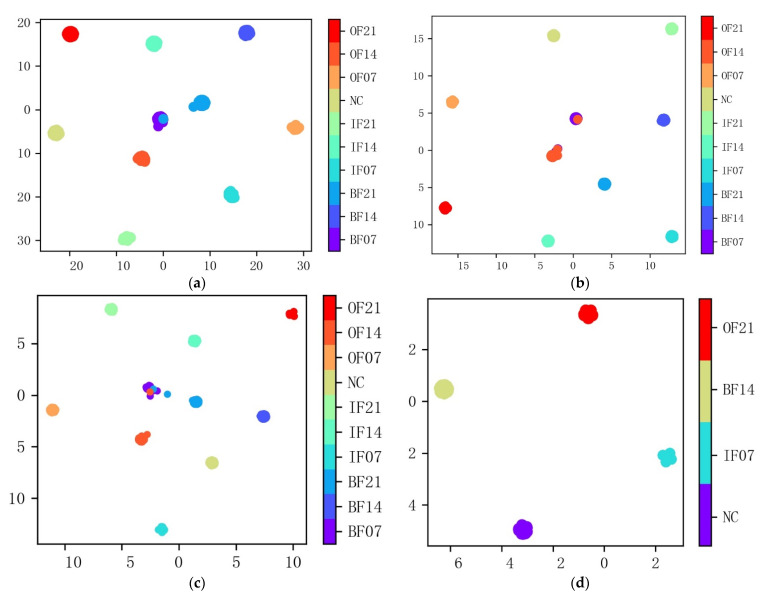
Feature visualization by t-SNE for each dataset: (**a**) Dataset A; (**b**) Dataset B; (**c**) Dataset C; (**d**) Dataset D.

In order to qualitatively illustrate the effectiveness of the proposed model and judge the separability of the data on the basis of the visualization of learned representation, nonlinear dimensionality reduction algorithm t-SNE was employed to project the data into a 2-dimensional space. Figure 8 shows the visualization results of the MTF-ResNet model for the 10- and 4-category datasets.

The model had powerful feature extraction and classification capability, samples of different fault types were almost perfectly separated, and samples within the same type were intuitively clustered. The results of feature visualization are consistent with the confusion matrices and demonstrate that the fault diagnosis problem can be successfully addressed by the proposed MTF-ResNet model.

**Figure 9:**
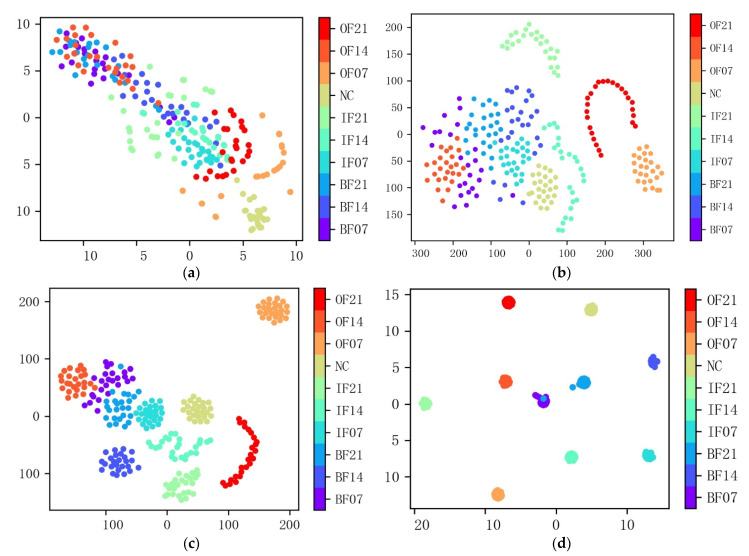
Feature visualization of different layers of the proposed MTF-ResNet. (**a**) First convolutional layer; (**b**) 13th convolutional layer; (**c**) 23rd convolutional layer; (**d**) fully connected layer.

To better understand the effect of convolutional layers of the model in fault diagnosis, the features extracted from the four convolutional layers are visually mapped into a two-dimensional distribution by t-SNE, as shown in Figure 9.

Figure 9a shows the distribution results of the first convolutional layer, the redundancy of the vibration signal itself makes it difficult to distinguish between the different fault types. From Figure 9b, the samples of IF21, OF21 and OF07 are separated out while the rest samples of different categories are mixed. After the 23rd convolutional layer, the output sample distribution significantly changed. Most of the samples are clustered in their respective regions, but there are still some samples that are not clustered and are scattered among the adjacent categories, as shown in Figure 9c. Results of the fully connected layer are shown in Figure 9d; all samples were separated out and then clustered into their regions except for the rolling ball fault samples, which had a certain degree of misclassification.

### 4.3. Model Performance with Different Residual Network Structures

In this section, the performance of the MTF-ResNet model with different residual network structures is investigated. The same 10-category dataset was adopted, and the encoded MTF images were applied as input in ResNet-18 and ResNet-50 for feature extraction and classification. The average classification accuracy of different residual structures is shown in Table 6. It is clear that the residual networks achieved good classification accuracy of over 94% for images of bearing fault signals converted by the Markovt transition field, and the model using ResNet-34 achieved better accuracy of over 4.67% and 2.16% than that of the models using ResNet-18 and ResNet-50, respectively.

### 4.4. Comparison with Other Methods

In recent years, much research has been conducted for rolling-bearing fault diagnosis problems. In order to further prove the superiority of the MTF-ResNet method proposed in this paper, we compared it with some commonly used methods. The detailed comparison results are shown in Table 7. As obtained from the experimental results, the method in [25] could achieve 100% testing accuracy, but the model was only validated for 4-category fault classification. The proposed method could achieve an average accuracy of 98.52% for 10-category datasets and 100% for 4-category dataset. Compared with the methods in [33,34,35,36], the proposed MTF-ResNet method could identify more fault types and improve classification accuracy.

## 5. Conclusions

In this work, we proposed a novel intelligent rolling-bearing fault diagnosis method based on the Markov transition field (MTF) and residual network. Encoding one-dimensional time-series signals into two-dimensional images by Markov transition field preserves the time dependence of the raw signals and discards the prior knowledge to set parameters during the conversion. On this basis, a residual network is applied to identify the fault types through image classification. Experiments conducted on the CWRU bearing dataset indicate that MTF-ResNet achieved prominent performance on the identification of rolling bearings faults with various degrees of severity and locations, the proposed model achieves an average accuracy of 100% and 98.52% in the 4- and 10-category datasets, respectively. Compared with other intelligent bearing-fault diagnosis methods, the proposed MTF-ResNet method offers a better performance of feature extraction and classification in the fault diagnosis.

While the MTF-ResNet method can achieve good performance for fault diagnosis, it has the disadvantage of requiring a longer training period than other shallow neural network-based methods do, as the residual network in this study was trained from scratch. Deep-learning algorithms are frequently hampered by a high computational burden. In further work, the transfer-learning approach, which showed promising results in reducing training time and computational cost [37], will be considered to be employed in machinery fault diagnosis tasks. In addition, further investigations into the effectiveness of the MTF-ResNet method should be carried out a wider variety of datasets, such as gear- and rotor-fault datasets.

## Figures and Tables

**Table 1:** Structure of residual networks.

Layer Name	ResNet-18	ResNet-34	ResNet-50	Output Size
Conv1	7 × 7, 64, stride 2			112 × 112
Conv2_x	3 × 3 max pool, stride 2			56 × 56
	$\left[\begin{array}{cc} 3\times 3, & 64\\ 3\times 3, & 64 \end{array}\right]\times 2$	$\left[\begin{array}{cc} 3\times 3, & 64\\ 3\times 3, & 64 \end{array}\right]\times 3$	$\left[\begin{array}{cc} \begin{array}{l} 1\times 1,\\ 3\times 3, \end{array} & \begin{array}{l} 64\\ 64 \end{array}\\ 1\times 1, & 256 \end{array}\right]\times 3$	
Conv3_x	$\left[\begin{array}{cc} 3\times 3, & 128\\ 3\times 3, & 128 \end{array}\right]\times 2$	$\left[\begin{array}{cc} 3\times 3, & 128\\ 3\times 3, & 128 \end{array}\right]\times 4$	$\left[\begin{array}{cc} \begin{array}{l} 1\times 1,\\ 3\times 3, \end{array} & \begin{array}{l} 128\\ 128 \end{array}\\ 1\times 1, & 512 \end{array}\right]\times 4$	28 × 28
Conv4_x	$\left[\begin{array}{cc} 3\times 3, & 256\\ 3\times 3, & 256 \end{array}\right]\times 2$	$\left[\begin{array}{cc} 3\times 3, & 256\\ 3\times 3, & 256 \end{array}\right]\times 6$	$\left[\begin{array}{cc} \begin{array}{l} 1\times 1,\\ 3\times 3, \end{array} & \begin{array}{l} 256\\ 256 \end{array}\\ 1\times 1, & 1028 \end{array}\right]\times 6$	14 × 14
Conv5_x	$\left[\begin{array}{cc} 3\times 3, & 512\\ 3\times 3, & 512 \end{array}\right]\times 2$	$\left[\begin{array}{cc} 3\times 3, & 512\\ 3\times 3, & 512 \end{array}\right]\times 3$	$\left[\begin{array}{cc} \begin{array}{l} 1\times 1,\\ 3\times 3, \end{array} & \begin{array}{l} 512\\ 512 \end{array}\\ 1\times 1, & 2048 \end{array}\right]\times 3$	7 × 7
	Average pool, fc, softmax			1 × 1

**Table 2:** Detailed parameters of the MTF-ResNet model.

Parameters	Value
Batch size	32
Optimizer	Adam
Lr	0.0001
Loss function	Category—cross-entropy

**Table 3:** Working conditions studied in this work.

Dataset	Motor Load (hp)	Motor Speed (r/min)
A	1	1772
B	2	1750
C	3	1730

**Table 4:** Composition of single working condition bearing fault data.

Fault Type	Fault Diameter (Inch)	Number of Samples	Label
BF07	0.007	660/25	0
BF14	0.014	660/25	1
BF21	0.021	660/25	2
IF07	0.007	660/25	3
IF14	0.014	660/25	4
IF21	0.021	660/25	5
NC	0	660/25	6
OF07	0.007	660/25	7
OF14	0.014	660/25	8
OF21	0.021	660/25	9

**Table 5:** Composition of bearing fault data under variable working conditions (Dataset D).

Fault Type	Fault Diameter (Inch)	Motor Load (hp)	Label
NC	0	0	0
IF07	0.007	1	1
BF14	0.014	2	2
OF21	0.021	3	3

**Table 6:** Average classification accuracy of different residual structures.

Network	Epoch	Accuracy (%)
ResNet-18	100	94.12
ResNet-34	100	98.52
ResNet-50	100	96.44

**Table 7:** Experimental results of different methods.

Methods	Categories	Accuracy (%)
VI-CNN [25]	4	100
STFT-CNN [33]	4	99.4
Compact 1D-CNN [34]	6	93.2
IDSCNN [35]	10	93.84
CNNEPDNN [36]	10	97.85
Proposed	4 10	100 98.52

## Data Availability

Case Western Reserve University Bearing Data https://engineering.case.edu/bearingdatacenter (accessed on 5 February 2022).
